# Do Participants With Different Patterns of Loss to Follow-Up Have Different Characteristics? A Multi-Wave Longitudinal Study

**DOI:** 10.2188/jea.JE20150015

**Published:** 2016-01-05

**Authors:** Nargess Saiepour, Robert Ware, Jake Najman, Peter Baker, Alexandra Clavarino, Gail Williams

**Affiliations:** 1 School of Public Health, The University of Queensland, Herston, Australia; 2 Schools of Social Sciences, The University of Queensland, St. Lucia, Australia; 3 School of Pharmacy, The University of Queensland, Woolloongabba, Australia

**Keywords:** patterns of loss to follow-up, longitudinal study, missing data, attrition, characteristics

## Abstract

**Background:**

To identify patterns of loss to follow-up and baseline predictors of each pattern.

**Methods:**

The Mater-University Study of Pregnancy collected baseline information for 7718 pregnant women who attended Mater Hospital in Brisbane, Australia, from 1981 through 1983. Follow-up data for 6753 eligible participants were collected at 6 months, 5 years, 14 years, 21 years, and 27 years after giving birth. Participants were partitioned into groups of ‘Always Responders’, ‘Returners’, ‘Leavers’, ‘Intermittents’, and ‘Never Responders’. Multinomial logistic regression was used to simultaneously compare baseline characteristics of the last four groups with ‘Always Responders’.

**Results:**

Being younger, less educated, having no partner, and living in rented housing were associated with being a ‘Returner’. Not owning housing, receiving welfare benefits, and being younger, less educated, not married, a smoker, an Aboriginal/Islander, and born in a non-English-speaking country were associated with being a ‘Leaver’, an ‘Intermittent’, or a ‘Never-responder’. Having higher mental health score and drinking before pregnancy were associated with being a ‘Leaver’ or an ‘Intermittent’. Being unemployed and not physically active were associated with being a ‘Leaver’ or ‘Never Responder’. The groups ‘Leavers’ and ‘Never Responders’ were the most different from the ‘Always Responders’. The group that was most similar to ‘Always Responders’ was the ‘Returners’.

**Conclusions:**

Patterns of loss to follow-up should be considered in the application of missing data techniques, where researchers make assumptions about the characteristics of those subjects who do not respond to assess the type of missing data. This information can be used to prevent individuals who are at high risk of dropping out of a study from doing so.

## INTRODUCTION

Missing data pose major methodological challenges to longitudinal studies.^1^^,^^2^ Data can be missing because participants may not have completed all items of a questionnaire or test measurement (item non-response), they may have skipped a whole phase (wave non-response), or they may have dropped out of the study (attrition).^3^

Cohort studies in particular are an important tool for examining causal relationships they aim to present results that are valid and representative of the reference population.^4^ However, attrition in cohort studies is unavoidable and sometimes considerable,^5^ with potentially deleterious effects on results that undermine the value of the study.^4^ If the probability of data being missing (loss to follow-up) is related to observed characteristics, attrition can produce a data set that is no longer representative of the population of interest.^6^ As a result, estimates based on such data may be subject to attrition bias.^7^ It is important to identify participants who are at greatest risk of becoming lost to follow-up in order to implement preventive strategies and to inform analytic strategies by making appropriate judgements about the nature of the missing data.^8^

In studies where participants can leave and then re-enter, loss to follow-up is not monotone, and the sample is dynamic over time, since many participants responding to a particular wave might have been missing in previous waves and vice versa.

Loss to follow-up can be thought of as a continuum. In a longitudinal study, one can distinguish more or less severe forms of loss to follow-up, where severity is manifested in terms of bias or other deleterious effects on study findings. Identifying factors associated with an individual participant’s pattern of response in long multiple-wave cohort studies can potentially provide valuable information, allowing researchers to assess whether those participants who return to a study (after being lost for at least one wave) can be informative about other missing participants.

Using data from a 27-year cohort study of pregnancy with almost complete baseline information, the Mater-University of Queensland Study of Pregnancy (MUSP), we aimed to identify: (i) the grouping corresponding to major patterns of loss to follow-up based on the history of response over five time points; and (ii) baseline predictors for these groups and the differences between participants belonging to each group.

## MATERIAL AND METHODS

The MUSP is an ongoing longitudinal study of pregnant women who attended the Mater Misericordiae Hospital in Brisbane, Australia during their pregnancy. The MUSP study has been previously described.^9^ The study began with collecting baseline information from 1981 through 1983 (Phase A). This information was collected for 7718 pregnant women who agreed to participate, out of a total of 7816 women who were approached. Follow-up phases were conducted for 6753 eligible participants. Eligibility criteria were as follows: a child discharged alive from the hospital who was not adopted prior to discharge, with completion of the baseline (during pregnancy) survey and a follow-up survey 5 days after giving birth. Follow-up data were collected on maternal and child demographics, lifestyle, and mental health at 6 months, 5 years, 14 years, 21 years, and 27 years after giving birth.^9^ Ethics approval was obtained from relevant committees at The University of Queensland and the Mater Misericordiae Hospital.

At each follow-up, participants were re-contacted using telephone and/or address contact details they had provided at baseline or the previous wave (including contact details of up to four relatives or friends). Participants were invited to attend an interview at the study hospital. Participants who could not attend an interview were sent a postal questionnaire. Those who agreed to an interview but were unable to travel to the study hospital were interviewed in their homes. A participant was defined as having responded to a particular survey wave if they were either interviewed in person or they completed the postal questionnaire. Any participant who actively withdrew from the study was not re-contacted at any further follow-up.

To identify the pattern of loss to follow-up, participants were partitioned into 5 different groups according to their history of response at the 6-month, 5-year, 14-year, 21-year, and 27-year follow-ups. ‘Always Responders’ were defined as participants who responded to all five waves. ‘Never Responders’ did not respond to any of the five waves. Intermittent responders were split into three groups: ‘Returners’, ‘Leavers’, and ‘Intermittent Responders’. ‘Returners’ missed at least the wave before returning to the study and responding to the rest of the follow-ups; ‘Leavers’ responded at least to the first wave, but did not respond to later waves, and ‘Intermittent Responders’ were participants who missed at least the first wave but after responding to the next wave continued to leave and return (ie, participants missed at least one wave after returning to the study).

A multinomial logistic regression model was used to simultaneously compare ‘Returners’, ‘Leavers’, ‘Intermittent Responders’, and ‘Never Responders’ with ‘Always Responders’. Age (13–19, 20–29, or 30–49 years), education (college or university; grade 10, 11, or 12; or primary school or less), marital status (married, living with partner, or no partner), ethnicity (Caucasian, Aboriginal/Islander, or others), country of birth (Australia, English-speaking country, or Non-English-speaking country), employment (yes [full time/part time], no), receipt of welfare benefits (yes or no), housing status (own, rent, or other), going to church (yes or no), physical activity (yes or no), smoking during pregnancy (yes or no), smoking before pregnancy (yes or no), drinking before pregnancy (yes or no), illicit drug use during pregnancy (yes or no), problems with the law (yes or no), and mental health scores (higher scores demonstrate poorer mental health) were examined in univariate multinomial logistic models, and a variable was included as a covariate for the multiple multinomial logistic model if it was significant at the *P* ≤ 0.1 level in the univariate analysis. Odds ratios (ORs) with 95% confidence intervals (CIs) for response were calculated using ‘Always Responders’ as the reference category.

## RESULTS

Women (*n* = 6753) were recruited at 5–38 weeks’ gestation and were aged between 13.2 and 46.9 years (median, 24.3 years). Of all participants, 6264 (95.3%) responded to the 6-month follow-up, 4843 (73.7%) responded to the 5-year follow-up, 4609 (70.1%) responded to the 14-year follow-up, 3715 (56.5%) responded to the 21-year follow-up, and 3558 (54.1%) responded to the 27-year follow-up.

When categorised according to pattern of loss to follow-up, there were 2561 (37.9%) ‘Always Responders’, 926 (13.7%) ‘Returners’, 2497 (37.0%) ‘Leavers’, 490 (7.3%) ‘Intermittent Responders’, and 279 (4.1%) ‘Never Responders’, with statistically significant differences in their characteristics.

Crude and adjusted ORs for predictors of different patterns of loss to follow-up are displayed in Table 1 and Table 2. After adjustment for all identified variables, the groups ‘Leavers’ and ‘Never Responders’ were the most different from the ‘Always Responders’ (the reference group). Almost all variables in the analysis were predictors of membership in these groups; only poorer mental health, not being Caucasian or identifying as Aboriginal/Islander, and being born in a non-English speaking country did not predict being a ‘Never Responder’. However, for the ‘Never Responders’, ORs were larger than for all other groups. The group that was most similar to the ‘Always Responders’ was the ‘Returners’; for this group, only age, education, and housing status were significant predictors. The ‘Intermittent Responders’ shared some characteristics with the ‘Returners’, ‘Leavers’, and ‘Never Responders’. For ‘Returners’, country of birth, employment, and physical activity were not predictors of being an ‘Intermittent Responder’; for ‘Leavers’, drinking before pregnancy and higher mental health score were associated with being an ‘Intermittent Responder’; and for ‘Never Responders’, not being Caucasian or identifying as Aboriginal/Islander and being born in a non-English speaking country were not predictors of being an ‘Intermittent Responder’.

**Table 1.** Baseline predictors (crude ORs) of different patterns of loss to follow-up^a^

Characteristic	*n*	Returner		Leaver		Intermittent		Never responder	
			
		OR	95% CI	OR	95% CI	OR	95% CI	OR	95% CI
Age, years									
13–19	1141	1.00		1.00		1.00		1.00	
20–29	4447	0.57	0.46, 0.71	0.54	0.46, 0.64	0.35	0.27, 0.45	0.30	0.22, 0.40
30–49	1165	0.48	0.37, 0.63	0.53	0.43, 0.64	0.39	0.28, 0.53	0.20	0.13, 0.31
Education									
College or university	1186	1.00		1.00		1.00		1.00	
Grade 10, 11 or 12	4294	1.44	1.18, 1.76	1.33	1.15, 1.54	1.60	1.21, 2.13	2.98	1.88, 4.73
Primary school or less	1219	1.60	1.23, 2.09	2.19	1.82, 2.64	2.67	1.91, 3.73	5.08	3.05, 8.44
Marital status									
Married	4971	1.00		1.00		1.00		1.00	
Living with partner	816	1.77	1.38, 2.26	2.17	1.81, 2.61	2.83	2.12, 3.76	5.08	3.67, 7.01
No partner	914	1.82	1.44, 2.30	2.10	1.76, 2.51	3.33	2.57, 4.32	4.38	3.17, 6.05
Ethnicity									
Caucasian	6033	1.00		1.00		1.00		1.00	
Aboriginal/Islander	253	2.27	1.41, 3.67	3.57	2.48, 5.13	4.26	2.61, 6.97	7.81	4.74, 12.88
Others	258	1.03	0.63, 1.67	2.36	1.75, 3.20	1.97	1.20, 3.23	2.13	1.16, 3.93
Country of birth									
Australia	4956	1.00		1.00		1.00		1.00	
English-speaking country	1012	1.04	0.84, 1.30	1.42	1.22, 1.66	1.07	0.75, 1.34	1.26	0.90, 1.78
Non-English-speaking country	705	1.04	0.79, 1.37	1.98	1.64, 2.38	1.45	1.05, 1.10	1.20	0.77, 1.86
Employment									
Yes (Full time/part time)	1865	1.00		1.00		1.00		1.00	
No	4834	1.24	1.05, 1.45	1.74	1.54, 1.97	1.65	1.32, 2.07	3.30	2.31, 4.71
Receiving welfare benefits									
No	5297	1.00		1.00		1.00		1.00	
Yes	1192	1.56	1.26, 1.94	2.33	1.99, 2.72	3.32	2.62, 4.21	4.48	3.39, 5.94
Accommodation									
Own	2843	1.00		1.00		1.00		1.00	
Renting	2777	1.73	1.46, 2.04	2.16	1.91, 2.45	2.91	2.31, 3.66	5.77	4.14, 8.05
Other	1049	1.72	1.37, 2.16	2.11	1.78, 2.50	3.99	3.03, 5.26	5.53	3.70, 8.25
Physical activity									
Active	3859	1.00		1.00		1.00		1.00	
Not active	2743	1.02	0.87, 1.19	1.43	1.28, 1.60	1.28	1.05, 1.56	1.85	1.44, 2.38
Smoking during pregnancy									
No	4083								
Yes	2599	1.03	1.11, 1.52	1.63	1.45, 1.83	1.97	1.62, 2.40	2.54	1.97, 3.26
Drinking Alcohol before pregnancy									
No	1661	1.00		1.00		1.00		1.00	
Yes	5041	0.98	0.82, 1.17	0.68	0.59, 0.77	0.80	0.64, 1.00	0.78	0.59, 1.03
Mental health^b^ (scores)	6451	1.28	1.09, 1.50	1.50	1.33, 1.68	2.02	1.69, 2.41	1.98	1.58, 2.48

CI, confidence interval; OR, odds ratio.

^a^‘Always Responders’ = 2561, ‘Returners’ = 926, ‘Leavers’ = 2497, ‘Intermittents’ = 490 and ‘Never responders’ = 279.

^b^Mental health was measured by a 10-item personal disturbance scale (DSSI/sAD) created using Mokken scaling.^10^

**Table 2.** Baseline predictors (adjusted ORs) of different patterns of loss to follow-up^a^

Characteristic	*n*	Returner		Leaver		Intermittent		Never responder	
			
		OR	95% CI	OR	95% CI	OR	95% CI	OR	95% CI
Age, years									
13–19	1141	1.00		1.00		1.00		1.00	
20–29	4447	0.72	0.56, 0.92	0.76	0.63, 0.93	0.67	0.50, 0.91	0.61	0.43, 0.88
30–49	1165	0.63	0.46, 0.87	0.72	0.56, 0.92	0.88	0.59, 1.30	0.49	0.28, 0.84
Education									
College or university	1186	1.00		1.00		1.00		1.00	
Grade 10, 11 or 12	4294	1.40	1.12, 1.74	1.22	1.04, 1.44	1.42	1.03, 1.94	2.70	1.58, 4.62
Primary school or less	1219	1.39	1.04, 1.87	1.64	1.33, 2.03	1.82	1.24, 2.66	3.12	1.72, 5.65
Marital status									
Married	4971	1.00		1.00		1.00		1.00	
Living with partner	816	1.32	0.99, 1.76	1.58	1.27, 1.96	1.58	1.12, 2.23	2.41	1.63, 3.55
No partner	914	1.40	1.02, 1.93	1.43	1.12, 1.83	1.45	1.00, 2.11	1.71	1.07, 2.73
Ethnicity									
Caucasian	6033	1.00		1.00		1.00		1.00	
Aboriginal/Islander	253	1.60	0.93, 2.74	2.53	1.70, 3.78	2.86	1.66, 4.93	4.66	2.62, 8.28
Others	258	1.01	0.56, 1.82	1.63	1.10, 2.41	1.32	0.66, 2.63	1.83	0.70, 4.75
Country of birth									
Australia	4956	1.00		1.00		1.00		1.00	
English-speaking country	1012	1.10	0.87, 1.39	1.60	1.34, 1.90	1.02	0.73, 1.43	1.60	1.10, 2.32
Non-English-speaking country	705	1.18	0.84, 1.67	1.70	1.33, 2.18	1.44	0.94, 2.19	1.07	0.57, 1.98
Employment									
Yes (Full time/part time)	1865	1.00		1.00		1.00		1.00	
No	4834	1.13	0.95, 1.35	1.38	1.20, 1.59	1.24	0.96, 1.61	2.10	1.42, 3.11
Receiving welfare benefits									
No	5297	1.00		1.00		1.00		1.00	
Yes	1192	1.12	0.87, 1.45	1.40	1.15, 1.70	1.57	1.17, 2.11	1.68	1.18, 2.38
Accommodation									
Own	2843	1.00		1.00		1.00		1.00	
Renting	2777	1.51	1.25, 1.82	1.60	1.39, 1.85	2.19	1.68, 2.87	3.33	2.26, 4.90
Other	1049	1.13	0.84, 1.54	1.22	0.97, 1.53	1.98	1.35, 2.90	2.00	1.18, 3.38
Physical activity									
Active	3859	1.00		1.00		1.00		1.00	
Not active	2743	1.03	0.87, 1.22	1.29	1.13, 1.47	1.14	0.91, 1.43	1.85	1.39, 2.46
Smoking during pregnancy									
No	4083	1.00		1.00		1.00		1.00	
Yes	2599	1.02	0.85, 1.21	1.37	1.19, 1.56	1.50	1.19, 1.90	1.51	1.12, 2.03
Drinking Alcohol before pregnancy									
No	1661	1.00		1.00		1.00		1.00	
Yes	5041	0.97	0.79, 1.19	0.74	0.63, 0.86	0.71	0.55, 0.93	0.81	0.58, 1.14
Mental health^b^ (scores)	6451	1.03	0.87, 1.24	1.17	1.02, 1.34	1.45	1.19, 1.78	1.28	0.99, 1.65

CI, confidence interval; OR, odds ratio.

^a^‘Always Responders’ = 2561, ‘Returners’ = 926, ‘Leavers’ = 2497, ‘Intermittents’ = 490 and ‘Never responders’ = 279.

^b^Mental health was measured by a 10-item personal disturbance scale (DSSI/sAD) created using Mokken scaling.^10^

## DISCUSSION

This study provides information on the characteristics of women from reproductive to post-reproductive ages who had various patterns of loss to follow-up in the MUSP cohort. There was almost complete ascertainment for all women at baseline, as recruitment occurred when the women were attending the hospital for their first antenatal visit.

Results show that people with different patterns of response have different characteristics. Women who owned their housing and who were older, married, and highly educated were more likely to be ‘Always Responders’. The three groupings of ‘Leavers’, ‘Intermittent Responders’, and ‘Never Responders’ were more similar to each other than to the ‘Returners’. The magnitude of associations increased from ‘Returners’ to ‘Leavers’, to ‘Intermittent Responders’, and to ‘Never Responders’, respectively. Most variables were associated with being a ‘Never Responder’. Being a ‘Returner’ was associated with younger age, having no partner, lower education, and living in rented housing. However, most of the other variables were predictors of being a ‘Leaver’, an ‘Intermittent Responder’, or a ‘Never Responder’. ‘Leavers’ and ‘Never Responders’ shared the most characteristics, and ‘Intermittent Responders’ had some similarities with ‘Leavers’ and some similarities with ‘Never Responders’. ‘Returners’ differed from ‘Always Responders’ in only few demographic variables, but ‘Leavers’, ‘Intermittent Responders’, and ‘Never Responders’ differed from ‘Always Responders’ in most characteristics.

In the present study, determinants of patterns of loss to follow-up were identified according to baseline characteristics. However, loss to follow-up is ascertained in later waves of the study, at which times the values of these determinants may have changed, leading to a change in the risk of attrition in later waves.^11^ The current study examined the predictors of patterns of loss to follow-up over multiple waves of a 27-year longitudinal study, while most past studies have covered a shorter time frame and have been limited to fewer waves.

Since MUSP recruited only women who were pregnant (ie, women of reproductive age) and who were attending that particular public hospital, the sample is likely to represent pregnant women of lower to middle socioeconomic status.

Different sources of loss to follow-up, such as loss of contacts, refusal, and ineligibility because of illness or death,^12^ may have different determinants.^13^ This study did not differentiate between attrition through non-contact and attrition through other sources. Since data were used from a cohort of relatively young women, attrition is less likely to be due to very poor health or death.^12^ Loss to follow-up in this study was more likely to be due to loss of contact (ie, failure to locate the participants) rather than refusal because the resources required to locate the least available respondents were never sufficient. This assumption is supported by the fact that, whenever we were successful in locating women lost to follow-up in previous waves, they usually agreed to return to the study.^9^ At the 27-year follow-up, a high proportion of traceable women who were lost to follow-up in previous waves agreed to return to the study. Of those who were lost at the 6-month, 5-year, 14-year, and 21-year follow-ups, 55%, 58%, 74%, and 77%, respectively, agreed to return to the study at the 27-year follow-up.

This information may be used to prevent individuals who are at high risk of dropping out of a study from being lost to follow-up. For instance, additional measures may be used to improve participation rates in these groups. Differences in characteristics of different responders can influence the results of a study if these are not properly considered while applying techniques that adjust for missing data.

### Conclusion

The question of whether the patterns of loss to follow-up matters in the analysis of missing data in cohort studies is an important issue that should receive appropriate consideration. Inconsistency in the predictors of different patterns of loss to follow-up suggests that history of response depends on the characteristics of participants, and this should be reflected when adjusting for missing data. This information can benefit researchers by informing strategies to reduce loss to follow-up, assess missing data, and apply techniques to account for missing data.^14^
